# SARS-CoV-2 Virus Culture and Subgenomic RNA for Respiratory Specimens from Patients with Mild Coronavirus Disease

**DOI:** 10.3201/eid2611.203219

**Published:** 2020-11

**Authors:** Ranawaka A.P.M. Perera, Eugene Tso, Owen T.Y. Tsang, Dominic N.C. Tsang, Kitty Fung, Yonna W.Y. Leung, Alex W.H. Chin, Daniel K.W. Chu, Samuel M.S. Cheng, Leo L.M. Poon, Vivien W.M. Chuang, Malik Peiris

**Affiliations:** The University of Hong Kong, Hong Kong, China (R.A.P.M. Perera, Y.W.Y. Leung, A.W.H. Chin, D.K.W. Chu, S.M.S. Cheng, L.L.M. Poon, M. Peiris);; United Christian Hospital, Hong Kong (E. Tso, K. Fung);; Centre for Health Protection, Hong Kong (D.N.C. Tsang);; Hospital Authority of Hong Kong, Hong Kong (V.W. M. Chuang);; Princess Margaret Hospital, Hong Kong (O.T.Y. Tsang)

**Keywords:** severe acute respiratory syndrome coronavirus 2, SARS-CoV-2, coronavirus, viruses, coronavirus disease, COVID-19, mild disease, viral RNA load, subgenomic RNA, virus culture, infectivity, respiratory infections, zoonoses, Hong Kong, China

## Abstract

We investigated 68 respiratory specimens from 35 coronavirus disease patients in Hong Kong, of whom 32 had mild disease. We found that severe acute respiratory syndrome coronavirus 2 and subgenomic RNA were rarely detectable beyond 8 days after onset of illness. However, virus RNA was detectable for many weeks by reverse transcription PCR.

Severe acute respiratory syndrome (SARS) coronavirus 2 (SARS-CoV-2) is causing a global pandemic and affecting global health and the world economy. Virus RNA might be detectable by reverse transcription PCR (RT-PCR) many weeks after clinical recovery (*1*,*2*), which affects the duration of isolation of patients. Similar findings were seen with SARS during 2003 (*3*). A large proportion of transmission occurs before and soon after onset of illness (*4*). However, the duration of contagiousness after the onset of clinical symptoms remains poorly understood. This duration is relevant to determining policy for discharge of patients from containment in hospitals.

Viral RNA detection by RT-PCR does not prove the presence of infectious virus; culture isolation of virus is a better indication of contagiousness. Recent studies on experimentally infected hamsters showed efficient transmission of SARS-CoV-2 to contact hamsters on day 1 after challenge when virus culture results were positive in nasal washes, but not at day 6 when nasal washes were culture negative, although viral load determined by RT-PCR was still high (>6.0 log_10_ RNA copies/mL) (*5*). Thus, virus culture might be a better surrogate for transmissibility.

We attempted virus isolation in 68 specimens from 35 patients in Hong Kong. Specimens were collected at different times after symptom onset to define the kinetics of virus isolation in upper respiratory specimens. Those specimens with a viral load >5 log_10_ were also examined for detection of subgenomic viral RNA (sgRNA).

## The Study

The study was approved by the Research Ethics Committee of the Kowloon West Cluster (reference No. KW/EX-20–039; 144–27) of the Hospital Authority of Hong Kong. We provide methods for virus nucleoprotein (N) gene copy number quantification (*6*), virus culture, and sgRNA detection of RT-PCR–confirmed coronavirus disease (COVID-19) patients (Appendix). Virus sgRNA was tested in specimens that had >5 log_10_ N gene copies/mL.

A total of 68 specimens from 35 patients were studied (Table 1; Appendix); patients with prolonged virus shedding (10 who remained virus RNA positive for >30 days) and patients readmitted because RT-PCR positivity was detected after discharge (n = 6) were oversampled (i.e., selected to make up a larger share of the survey sample than is performed for the patient population). Patient age ranged from 17 to 75 years (median 38 years); 23 were male and 12 female (Table 1). Specimens submitted for virus culture were nasopharyngeal aspirates and throat swab specimens (n = 46), nasopharyngeal aspirates (n = 2), nasopharyngeal swab specimens and throat swab specimens (n = 4), nasopharyngeal swab specimens (n = 3), sputum (n = 11), and saliva (n = 2). The duration after onset of illness to specimen collection ranged from 1 to 67 days.

**Table 1:** Comparison of patients and clinical respiratory specimens that were positive or negative by culture for severe acute respiratory syndrome coronavirus 2 and duration of illness for patients with mild coronavirus disease, Hong Kong*

Characteristic	Culture positive, n = 16	Culture negative, n = 52	Total, n = 68	Statistical significance
Patients, n = 35				
Asymptomatic	1	2	3	ND
Median age (range), y	39 (21–73)	38 (17–75)	38	ND
Concurrent condition	5	4	9	ND
Clinical specimens				
Median log_10_ viral load/mL†, n = 68	7.5	3.8		p<0.0001 by Mann- Whitney test
Viral load log_10_, range, n = 68				
7.0–9.5	12 (75)	5 (10)	17 (25)	p = 0.018 by Fisher exact test
6.0–6.99	3 (19)	8 (15)	11 (16)	
5.0–5.99	1 (6)	6 (12)	7 (10)	
<5.0	0	33 (63)	33 (49)	
Days after onset of illness when sample was collected, n = 68				
1–2	8 (53)	7 (13)	15 (22)	p = 0.00001 by Fisher exact test
3–8	8 (35)	15 (29)	23 (34)	
9–67	0	30‡ (58)	30 (44)	
Days after onset of illness when sample was collected from patients without or before antiviral treatment, n = 42				
1–2	8 (50)	7 (27)	15 (36)	p = 0.01 by Fisher exact test
3–8	8 (50)	9 (35)	17 (40)	
9–67	0	10 (38)	10 (24)	

*Values are no. (%) patients unless indicated otherwise. ND, not determined. †Viral load below limit of detection for the assay was scored as 1 log_10_/mL. ‡Six of these patients were readmissions because of detection of virus RNA after discharge from isolation.

**Figure 1:**
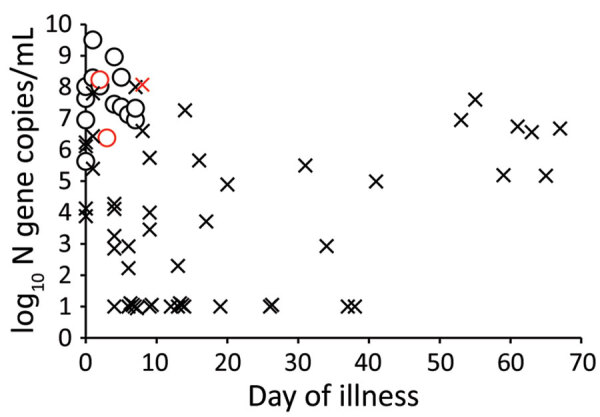
Severe acute respiratory syndrome coronavirus 2 RNA load, virus culture, and days after onset of illness in respiratory specimens and duration of illness for patients with mild coronavirus disease, Hong Kong. ¡ indicates samples positive by virus culture and × indicates samples negative by virus culture. Red indicates 2 critically ill patients and 1 patient who died; black indicates mild, moderate, or asymptomatic infections. The limit of detection of the viral N gene RNA was 1 log_10_ copies/mL; undetectable virus load is indicated as the limit of detection. N, nucleoprotein.

Virus was isolated from 16 specimens for 16 patients. The median age of the culture-positive patients was 39 years and of the culture-negative patients was 38 years. SARS-CoV-2 N gene copy number in the specimens overall ranged from 9.5 log_10_ copies/mL to undetectable (limit of detection 10 copies/mL) (Figure 1). The median viral load in culture-positive samples was 7.5 log_10_ copies/mL and in culture-negative samples was 3.8 log_10_ copies/mL (p = 0.00001) (Table 1).

Virus was isolated from 12 of 17 specimens with viral loads ≥7.0 log_10_ copies/mL, 3 of 11 specimens with viral loads 6.0–6.99 log_10_ copies/mL, 1 of 7 specimens with viral loads 5.0–5.99 log_10_ copies/mL, and 0 of 33 specimens viral loads <5 log_10_ copies/mL.

**Figure 2:**
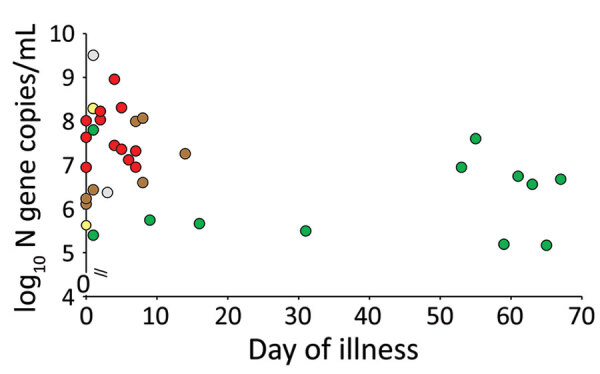
Severe acute respiratory syndrome coronavirus 2 viral RNA load, virus culture, and subgenomic virus RNA (sgRNA) in relation to days after onset of illness for patients with mild coronavirus disease, Hong Kong. Red indicates culture and sgRNA positive, green indicates culture and sgRNA negative, yellow indicates culture positive and sgRNA negative, brown indicates culture negative and sgRNA positive, and gray indicates culture positive and no sgRNA data (because of insufficient specimen).

The sgRNA provides evidence of replicative intermediates of the virus, rather than residual viral RNA. Detection of virus sgRNA was attempted for 33 of the 35 the clinical specimens that had viral loads >5.0 log_10_ virus genome copies/mL; 2 specimens had insufficient specimen for this testing. Of 33 specimens tested for sgRNA and by virus culture, both tests showed positive results for 12 (36.4%) specimens, both tests showed negative results for 12 (36.4%), sgRNA showed positive results and culture was negative for 7 (21.2%) specimens, and culture was positive and sgRNA showed negative results for 2 (6.1%) samples (Cohen κ 0.467, p = 0.005 against κ 0) indicating a moderate agreement between virus culture and sgRNA detection. Virus sgRNA was detectable in 18 (81.8%) of 22 specimens collected <8 days after symptom onset and in 1 (9.1%) of 11 specimens collected >9 days after onset of disease (p = 0.0003 by χ^2^ test with Yates correction) (Figure 2). We also provide culture and sgRNA results stratified by specimen type (Table 2).

**Table 2:** Culture and sgRNA results stratified by specimen type for patients with mild coronavirus disease, Hong Kong*

Specimen	Investigation	Days after onset of illness, no. positive/no. tested				
		1–2	3–8	9–14	15–21	>21
NPA + TS	Culture	8/14	2/14	0/8	0/2	0/8
	sgRNA	8/11	4/4	0/1	0/0	0/4
NPA	Culture		2/2			
	sgRNA		1/1			
NPS + TS	Culture		0/2	0/2		
	sgRNA					
NPS	Culture	1/1	1/1			0/1
	sgRNA	1/1	1/1			
Sputum	Culture		0/1	0/2	0/8	
	sgRNA		1/1	1/1	0/5	
Saliva	Culture		2/2			
	sgRNA		2/2			

*Blank cells indicate that no specimens were collected at that time point. NPA, nasopharyngeal aspirate; NPS, nasopharyngeal swab; sgRNA, subgenomic viral RNA: TS, throat swab.

We conducted a subset analysis for 42 specimens collected from patients who did not receive antiviral drugs or specimens that were collected before antiviral therapy (Table 1). This sample included all 16 specimens that were culture positive and 18 of 19 specimens that were sgRNA positive. The main conclusions remained unchanged. Median viral RNA load in culture-positive specimens was 7.54 log_10_ genome copies/mL and in culture-negative specimens was 4.0 log_10_ genome copies/mL (p<0.00001 by Mann-Whitney 2-tailed U test). Of the 16 culture positive specimens, 15 (94%) had viral RNA load >6 log_10_ copies/mL (p<0.01 by Fisher exact test). All of them were collected within the first 8 days of illness (p = 0.01 by Fisher exact test ) (Table 1). However, the duration of illness in this subset of specimens was limited to 31 days. Five specimens with viral load >6 log_10_ virus N gene copies/mL collected >50 days after onset of illness were negative by virus culture and virus sgRNA, but all of these patients had received antiviral therapy.

## Conclusions

For a cohort of patients with predominantly mild COVID-19, our findings suggest that virus isolation and sgRNA detection were positive within the first 8 days after onset of illness and mainly for specimens with >6 log_10_ virus N gene copies/mL of clinical specimen. We did not carry out serologic testing in parallel with viral culture, but data on a larger cohort of patients in Hong Kong showed that most patients had detectable virus neutralizing antibodies after day 9 of illness (*7*). Two other studies of virus culture for mildly ill or moderately ill patients showed virus culture was only successful within the first 9 days after onset of illness (*8**,**9*). Patients who are severely ill and immunocompromised might shed infectious virus for much longer periods (J.J.A. van Kampen, Erasmus University Medical Center, pers. comm., 2020 Jun 9), and this shedding might also be prolonged by corticosteroid therapy.

The World Health Organization has recently amended its guidelines for releasing COVID-19 patients from isolation (i.e., 10 days after symptom onset and >3 additional days without symptoms), but these guidelines do not distinguish between mild and severely ill patients (*10*). Our findings suggest that patients with mild or moderate illness might be less contagious 8 days after symptom onset. Mildly ill patients who have clinically recovered and are not immunocompromised might be discharged from containment >9 days after symptom onset, as long as they are not being discharged into settings that contain other highly vulnerable persons (e.g., old age care homes). 

Appendix Additional information on SARS-CoV-2 virus culture and subgenomic RNA for respiratory specimens from patients with mild coronavirus disease.
