# Supplementary material for: SARS-CoV-2 Virus Culture and Subgenomic RNA for Respiratory Specimens from Patients with Mild Coronavirus Disease
Source: Emerg Infect Dis. 2020 Nov;26(11):2701–4. doi: 10.3201/eid2611.203219 (PMC7588524; doi:10.3201/eid2611.203219)
Supplement: Appendix — Additional information on SARS-CoV-2 virus culture and subgenomic RNA for respiratory specimens from patients with mild coronavirus disease. [file 20-3219-Techapp-s1.pdf]

Article DOI: https://doi.org/10.3201/eid2611.203219

SARS-CoV-2 Virus Culture and Subgenomic RNA for Respiratory Specimens from Patients with Mild Coronavirus Disease

Appendix

Methods

Patients

Patients were given a diagnosis of coronavirus disease by reverse transcription PCR (RT-PCR) at individual hospitals. Follow-up specimens were sent to the School of Public Health, The University of Hong Kong, for virus culture and determination of viral RNA load. Specimens were transported and stored at 4°C until they were processed.

Nine patients had underlying concurrent conditions. None of the patients were immunocompromised. Three patients were asymptomatic; 29 patients had mild clinical illness (mild influenza-like illness symptoms not requiring supplemental oxygen, or requiring <3 L/min); 2 patients were in critical condition (intubated, required extracorporeal membrane oxygenation, or in shock); and 1 patient died.

Quantification of Virus Nucleoprotein Gene Copy Number

RNA was extracted from specimens by using the QIAamp Viral RNA Extraction Kit (QIAGEN, https://www.qiagen.com) according to the instructions of the manufacturer and tested by using an RT-quantitative PCR targeting the nucleoprotein (N) gene of severe acute respiratory syndrome coronavirus 2 (*1*). Absolute copy number was quantitated. A cloned plasmid DNA extract carrying a DNA insert of the N gene was prepared and used for determination of DNA copy number. The copy number control plasmid DNA with serial dilutions were included in each RT-quantitative PCR to construct a standard curve to correlate cycle threshold values and gene copy number of samples. Virus gene copy number per milliliter was calculated by adjusting for the dilution effect of nucleic acid extraction.

Virus Culture

Vero E6 cells (ATCC-ORL-1586) were seeded at a cell count of 150,000 cells/well into 24-well tissue culture plates (TPP Techno Plastic Products, https://www.tpp.ch) sufficient to give a subconfluent cell monolayer after incubation for 24 hours in a CO_2 incubator. The culture medium was removed and 125 µL of the clinical specimen in virus transport medium diluted 1:1 in Dulbecco modified Eagle medium (GIBCO, https://www.thermofisher.com) containing 2% fetal calf serum (GIBCO) was inoculated in into 2 wells. After 2 hours incubation in a CO_2 incubator at 37°C, 1 mL of Dulbecco modified Eagle medium containing 2% fetal calf serum was added the wells. The plates were incubated at 37°C in a CO_2 incubator. A sample (100 µL) of supernatant was sampled for a quantitative real-time RT-PCR at 0 and 72 hours postinoculation. At 72 hours, cells were scraped into the supernatant and transferred onto fresh cells in 24-well plates, followed by refeeding the cells with fresh culture medium and monitoring for an additional 72 hours. A final aliquot was collected for quantitative real-time RT-PCR. Cells were observed for cytopathic effect daily and harvested for passage if 25%–50% of cells showed a cytopathic effect.

Detection of Subgenomic mRNA by RT-PCR

RNA was extracted from samples by using the QIAamp Viral RNA Mini Kit (QIAGEN). The method was modified from published methods to detect coronavirus subgenomic mRNA (*2*). Purified RNA was reverse transcribed by using SuperScript II (ThermoFisher Scientific, https://www.thermofisher.com) and a severe acute respiratory syndrome coronavirus 2–specific primer (WHSA-29950R: 5′-TCTCCTAAGAAGCTATTAAAAT-3′). The complementary DNA obtained was subjected to PCR (40 cycles for 94°C for 30 s, 56°C for 30 s, and 72°C for 1.5 min. a condition optimized for amplifying small subgenomic mRNA) and AmpliTaq Gold DNA Polymerase (ThermoFisher Scientific) with primers (WHSA-00025F: 5′-CCAACCAACTTTCGATCTCTTGTA-3′ and WHSA-29925R: 5′-ATGGGGATAGCACTACTAAAATTA-3′). The PCR products were then subjected to 1% agarose gel electrophoresis.

Results

Patient concurrent conditions included diabetes mellitus, hypertension, ischemic heart disease, atrial fibrillation, chronic obstructive airways disease, carcinoma of the lung, mild

renal dysfunction, and chronic hepatitis B. For 3 asymptomatic patients, days after onset was estimated as days after first detection by RT-PCR. A total of 14 patients were sampled sequentially: 2–6 samples were collected from each patient at 3–4 day intervals. A total of 12 patients received no antiviral therapy, and 23 received lopinavir/ritonavir alone (n = 1) or in combination with ribavirin (n = 9), ribavirin and interferon β (n = 10), interferon β (n = 2), and ribavirin, β interferon, and tocilizumab (n = 1). Doses and regimens have been described (*3*). Duration of treatment was ≤14 days. Thus, antiviral drugs were not in use beyond day 30 of illness.

Of the 11 lower respiratory tract samples (sputa) collected, none of them yielded virus isolates, although 6 of them had viral N gene copies >6.0 $\log_{10}$/mL. Two them were collected ≤8 days of illness onset.

Corticosteroid therapy was used for 5 patients overall; 3 had critical conditions or died. Virus was isolated from 4 of these patients, and sgRNA was detected in 3 of them. For all patients, specimens were collected before commencement of corticosteroid therapy.

References

1. Chu DK, Pan Y, Cheng SM, Hui KP, Krishnan P, Liu Y, et al. Molecular diagnosis of a novel coronavirus (2019-nCoV) causing an outbreak of pneumonia. Clin Chem. 2020;66:549–55. PubMed https://doi.org/10.1093/clinchem/hvaa029

2. Simons FA, Vennema H, Rofina JE, Pol JM, Horzinek MC, Rottier PJ, et al. A mRNA PCR for the diagnosis of feline infectious peritonitis. J Virol Methods. 2005;124:111–6. PubMed https://doi.org/10.1016/j.jviromet.2004.11.012

3. Hung IF, Lung KC, Tso EY, Liu R, Chung TW, Chu MY, et al. Triple combination of interferon beta-1b, lopinavir-ritonavir, and ribavirin in the treatment of patients admitted to hospital with COVID-19: an open-label, randomised, phase 2 trial. Lancet. 2020;395:1695–704. PubMed https://doi.org/10.1016/S0140-6736(20)31042-4
